# Heritability of *P. falciparum* and *P. vivax* Malaria in a Karen Population in Thailand

**DOI:** 10.1371/journal.pone.0003887

**Published:** 2008-12-08

**Authors:** Waraphon Phimpraphi, Richard Paul, Bhee Witoonpanich, Chairat Turbpaiboon, Chayanon Peerapittayamongkol, Chalisa Louicharoen, Isabelle Casademont, Sumalee Tungpradabkul, Srivicha Krudsood, Jaranit Kaewkunwal, Thanyachai Sura, Sornchai Looareesuwan, Pratap Singhasivanon, Anavaj Sakuntabhai

**Affiliations:** 1 Institut Pasteur, Laboratoire de la Génétique de la réponse aux infections chez l'homme, Paris, France; 2 Department of Tropical Hygiene, Faculty of Tropical Medicine, Mahidol University, Bangkok Thailand; 3 Department of Biochemistry, Faculty of Science, Mahidol University, Bangkok, Thailand; 4 Department of Medicine, Faculty of Medicine Ramathibodi Hospital, Mahidol University, Bangkok, Thailand; 5 Department of Clinical Tropical Medicine, Faculty of Tropical Medicine, Mahidol University, Bangkok, Thailand; University of Oxford, United Kingdom

## Abstract

The majority of studies concerning malaria host genetics have focused on individual genes that confer protection against rather than susceptibility to malaria. Establishing the relative impact of genetic versus non-genetic factors on malaria infection and disease is essential to focus effort on key determinant factors. This relative contribution has rarely been evaluated for *Plasmodium falciparum* and almost never for *Plasmodium vivax*. We conducted a longitudinal cohort study in a Karen population of 3,484 individuals in a region of mesoendemic malaria, Thailand from 1998 to 2005. The number of *P. falciparum* and *P. vivax* clinical cases and the parasite density per person were determined. Statistical analyses were performed to account for the influence of environmental factors and the genetic heritability of the phenotypes was calculated using the pedigree-based variance components model. The genetic contribution to the number of clinical episodes resulting from *P. falciparum* and *P. vivax* were 10% and 19% respectively. There was also moderate genetic contribution to the maximum and overall parasite trophozoite density phenotypes for both *P. falciparum* (16%&16%) and *P. vivax* (15%&13%). These values, for *P. falciparum*, were similar to those previously observed in a region of much higher transmission intensity in Senegal, West Africa. Although environmental factors play an important role in acquiring an infection, genetics plays a determinant role in the outcome of an infection with either malaria parasite species prior to the development of immunity.

## Introduction

Human malaria is caused by four species of erythrocyte-invading plasmodial parasites, of which *Plasmodium falciparum* and *Plasmodium vivax* are the most common. These two species co-exist in many parts of the world including Thailand. Despite major attempts over the past century to control malaria, this infection remains the most important human parasitic disease worldwide. *P. falciparum* is estimated to cause about half a billion episodes per year [1], and there are hundreds of millions of cases due to *P. vivax*, *P. malariae* and *P. ovale*. Malaria parasites are becoming increasingly drug resistant and despite considerable effort over the last decade, there is as yet no sight of an effective vaccine.

Human genetic resistant factors for malaria were first discovered half a century ago, largely as a result of Haldane's insight that malaria was the likely evolutionary driving force behind common erythrocyte variants in tropical populations [2]. The high gene frequency of the sickle cell mutation (HbS) in Africa, which confers protection against severe malaria in heterozygotes but causes fatal sickle cell disease in homozygotes, illustrates the powerful selective pressure exerted by malaria on the human genome. Since, β -globin, and several other genes and genetic variants have been shown to be involved in the protection against or susceptibility to severe malaria, including α-globin, HLA and several cytokine loci [3]. Most of the protective variants are thought to have emerged in populations living in regions endemic for malaria as a result of the high selection pressure exerted by the parasite [2], [4]–[6]. In addition to a demonstrated implication of such candidate genes, family-based segregation and linkage analyses have repeatedly identified the importance of the chromosomal region 5q31-33 in the control of parasite density, with heritability exceeding 40% [7]–[11]. This region contains a cluster of cytokines, which may represent strong candidate genes for control of malaria infection, but the causative gene(s) and variant(s), remain to be identified. More recently, a genome-wide linkage study found locus-specific heritability on chromosome 13q for *P. falciparum* parasite density and chromosome 10p for the number of malaria episodes of approximately 35% [12]. This was suggested to support the conclusion that susceptibility and resistance to infectious diseases be governed by few major genes rather than many minor genes [13].

The ultimate goal of genetic epidemiology is to identify critical molecular pathways of pathogenesis and immunity, thereby generating novel strategies for treatment and prevention. Yet, both *Plasmodium* infection and malaria disease are well known to be strongly influenced by environmental factors. In addition, there are human non-genetic factors such as age and gender that also have a major effect on both incidence and outcome of infection. Establishing the relative impact of genetic versus non-genetic factors on infection and disease is essential to focus effort on key determinant factors. This relative contribution has been rarely evaluated [14]–[16], largely because of the difficulty in estimating the overall contribution of genetic factors (heritability, *h^2^*) in infection and disease. Estimation of heritability requires reliable information on family structure and sufficient data on well-defined malaria related phenotypes all the while controlling for heterogeneity in both the human genetic background and the force of infection. Longitudinal community-cohort studies are thus required.

In this study, we have estimated the contribution of host genetic factors to malaria relative to environmental factors in a Karen population located near the Thai-Myanmar border, which is an endemic area for *P. falciparum* and *P. vivax* malaria in Thailand.

## Materials and Methods

### Study site and family data

A community-based cohort study was carried out from June 1998 to May 2005. The study was conducted in a mountainous area of Suan Phung district, Ratchaburi province, Thailand. Suan Phung is a small district situated near the Thai-Myanmar border surrounded by the long Tanaosri ranges on the western side. The Tanaosri subdistrict is located at the southern part of Suan Phung, approximately 163 km west of Bangkok. Suan Phung has a total population of 5,368 living in 7 hamlets, of which 3,484 villagers of all ages participated in the study. This community is made up of a group of 4 closely related ethnic groups, the majority of which are Karen (85%), some Thai (14%) and the rest are Mon and Burmese (1%). The project protocol and objectives were explained to the population and informed consent was individually obtained from all subjects either by signature or by thumbprint on a voluntary consent form written in Thai, the local language. Such consent was obtained in the presence of the school director, an independent witness. For very young children, parents or designated tutors signed on their behalf. The recruitment procedure has been previously detailed [17]. Ethical permission for the study was granted by the Ethical Committee of the Ministry of Public Health of Thailand.

Family structures were constructed by using a questionnaire, interviewing each individual or key representatives of the household to obtain both demographic information such as birth date, age, sex, day of fever, and genetic relationships between children, their parents, and sometimes their grandparents or non-relatives in the same household, and other households. Pedigree structures were checked in 928 individuals, from whom we obtained DNA samples, by identity by state allele sharing of each relative pair from genotyping results of 400 microsatellite markers used during genome screening using IBS_check program (Heath, unpublished). There was <5% discordance including labelling errors. Pedigrees were modified accordingly.

The total pedigrees are comprised of 2,427 individuals, including absent or deceased relatives. There were 238 independent families containing 603 nuclear families; the majority are 2 generation-families with family size range from 3 to 958 (Table 1). For estimation of heritability, we used information from families that had at least 2 members with the phenotypes of interest, which varied according to occurrence of the diseases. The number of families and relative pair counts for each phenotype category are shown in Table 2.

**Table 1:** Family statistics.

Individuals characteristics		
Sex: M/F ratio	1.03	
Age median (min-max) yrs	14 (0–92)	
Age group	N	%
<1	52	2.0
1–4	332	13.0
5–9	429	16.9
10–14	370	14.5
15–24	494	19.4
25–39	513	20.2
40–59	282	11.1
60+	73	2.9

Family size:	Number of Pedigrees
3	39
4	50
5	40
6	39
7	23
8	10
9	11
10	5
11	3
12	3
14	3
15	3
16	1
17	3
19	1
20	1
28	1
38	1
958	1

Number of Generations	Number of Pedigrees
2	192
3	42
4	4

**Table 2:** Summary of samples used in epidemiological and genetic analyses.

		A. Clinical Phenotypes	B. Parasite Biological Phenotypes	B. Parasite Biological Phenotypes
			*Plasmodium falciparum*	*Plasmodium vivax*
Epidemiological analyses	Data points	19,162	1,795	975
	Individuals	2,545	949	517
Genetic analyses	Individuals	2,018 (832)	857 (371)	470 (232)
	Independent families	233	187	136
	Sib-pairs	1,950 (977)	551 (299)	202 (133)
	Halfsib-pairs	395 (292)	113 (86)	31 (26)
	Cousin-pairs	2,414 (2,307)	554 (544)	363 (362)
	Parent-Child-pairs	2,324 (1,110)	479 (267)	88 (49)
	Grandparent-pairs	849 (676)	117 (100)	43 (43)
	Avuncular-pairs	2,123 (1,859)	452 (420)	155 (140)

For epidemiological analyses, presented are the number of data points (observations and calculated mean/max values) analyzed for each phenotype category, the corresponding number of individuals implicated and hence residual values generated. For genetic analyses, presented are the number of these individuals for whom pedigree information was available and thus the number of independent families and relative pairs count for each phenotype in the heritability analyses. In parentheses, the number belonging to the large complex family.

### Malaria epidemiology

The epidemiology of malaria in this site has been described elsewhere [17]. Briefly, the incidence of malaria is highly seasonal with annual peaks in May-June and decreased over the duration of the study from 141 per 1000 person-years in 1999 to 57 in 2004 for *P. falciparum* and from 79 to 28 for *P. vivax*. *P. falciparum* prevalence rates varied from 1–7% seasonally and from 1–4% for *P. vivax*. There was good concordance in the proportion of fevers that were found to be positive for malaria parasites and the fraction of fevers attributable to malaria. Thus, in this site, virtually all infections lead to febrile episodes. Peak incidence occurred in an earlier age group (5–9 years old) for *P. vivax* than for *P. falciparum* (10–15 years old). Parasite densities of either species peaked in the <10 years old age group. Bed nets had no effect on incidence rates.

### Data Collection

The installation of a health clinic enabled passive case detection of malaria episodes and a record of non-malaria presentation. We defined clinical malaria episodes as measured fever (axillary temperature>37.5 °C) or fever-related symptoms including headache, back pain, chills, myalgia, nausea and vomiting associated with a slide positive for blood-stage trophozoite *P. falciparum*, *P. vivax, P. malariae* and *P. ovale* parasites at any density. To determine the correct number of clinical episodes, consecutive presentation at the clinic with blood-stage malaria parasite of the same species within 30 days after treatment of the initial infection was considered as the same episode. Consecutive presentation with non-malaria fever (or aforementioned symptoms) within 7 days following first presentation was likewise considered as the same episode. In addition, initial parasite negative visits were considered as part of a malaria episode if followed by a parasite-positive visit within the subsequent two days. All positive malaria cases were treated with appropriate antimalarial treatment according to the recommendation of the Malaria Division, Ministry of Public Health, as previously described [17]. Self-treatment is considered to be rare in the study area, because the only other access to treatment is a government clinic with which the study has good communication concerning malaria treatment of the study site population. In all cases parasite positivity was established as follows. Thick and thin blood films were prepared and stained by 3% Giemsa stain. Blood films were examined under an oil immersion objective at ×1000 magnification by the trained laboratory technicians and 200 thick film fields were examined before films were declared negative. Parasite species were identified on thin films and densities (per μl) were calculated from thick film by establishing the ratio of parasites to white blood cells (WBC) after at least 200–500 WBCs had been counted and then multiplying the parasite count by 8,000, the average WBC count per μl of blood.

### Disease phenotypes

Two categories of malaria phenotypes were considered in the study.

#### ***A. Clinical phenotypes.***

We studied the phenotypes related to clinical malaria episode in 2 different ways. (1) As the number of clinical visits that were positive for *P. falciparum* (Acronym PFA) or *P. vivax* (PVA) or which were negative i.e. number of non-malaria fever visits (Acronym NMF), (2) The proportion of visits that was positive for *P. falciparum* (Acronym PPFA) or *P. vivax* (Acronym PPVA). Using proportion in addition to number is an attempt to take into consideration differing tendency to visit the clinic; although virtually all malaria parasite infections are symptomatic, individuals who tend to visit the clinic very frequently may present with the few asymptomatic infections. In addition, this measure provides an independent measure that implicitly takes into account the time in the study.

#### ***B. Parasite biological phenotypes.***

For each species, we studied 2 different phenotypes related to parasite density during malaria clinical attacks. (1) Maximum trophozoite density (Acronym mx-PFD for *P. falciparum* and mx-PVD for *P. vivax*), (2) All trophozoite parasite densities excluding zero parasite density values i.e. excluding uninfected individuals (Acronym PFD for *P. falciparum* and PVD for *P. vivax*). Maximum density was chosen to assess whether there were individual human differences in the control of parasite density independently of parasite genotype. “All densities” was chosen to consider overall variation in the human-parasite interaction, taking into account variation due to parasite genotype.

### Data analyses for malaria and disease phenotypes

Table 3 gives a summary of the analyses and variables used for each phenotype. Statistical analyses and model fitting were conducted using the statistical package Genstat 7.1. All individuals in the study protocol were included in the analyses, irrespective of whether their family structure was known. From 3,484 participants, 939 never came to the clinic during the observation period, and were, therefore, excluded from the analyses. Factors influencing the maximum parasite density of either *P. falciparum* or *P. vivax* were analysed by fitting a Generalized Linear Model (GLM) with a Poisson error structure (loglinear regression). For phenotypes with repeated measures for the same individual (i.e. all phenotypes except maximum parasite density), a Generalized Linear Mixed Model (GLMM) was fitted with individual person as a factor in the random model. For number of episodes (non-malaria, *P. falciparum* or *P. vivax*) and parasite density, a Poisson error structure was implemented, thus yielding a loglinear regression. For proportion of visits that were positive for either species, a binomial error structure was implemented (thus a logistic regression). Explanatory factors include year (1998-2005), month, hamlet (7), and gender, with age factored into eight groups (<1, 1–4, 5–9, 10–14, 15–24, 25–39, 40–59 and 60+ years of age). Age was additionally refitted as a factor with two groups (<15 and ≥15 years of age), and as a continuous variable, but both proved less explanatory (lower adjusted r^2^) than age defined as eight groups. Initially a full model including all interactions possible was fitted and then the model was refined to include only statistically significant parameters and interactions. Inclusion of such interaction terms was considered important as it has been reported on numerous occasions that gender and age-specific behaviour can influence exposure and this can vary locally depending on the nature of the environment. Date was only considered as a main effect and not as an interaction with the other variables (age, gender, hamlet). The seasonality of malaria transmission in this region varies annually and thus month was considered only via its interaction with year (i.e. as a factor month-year). Analysing using date as a factor with just four levels (trimesters) proved less explanatory (lower adjusted r^2^). Because the data were over-dispersed a dispersion parameter was estimated. F-statistics in the GLM and Wald statistics, which approximate to a χ^2^ distribution, in the GLMM were established. In the analyses of the number of non-malaria or parasite species episodes, the time in the study was added as a weight; 89% of individuals were recruited from the outset and 97% by the end of the first year. In the analyses of maximum parasite density, the number of parasite density data points per individual was used as a weight.

**Table 3:** Percentage of variation in *P. falciparum* (Pf) and *P. vivax* (Pv) phenotypes explained by environmental factors (and their interactions) in the statistical analyses.

Phenotype	Acronym	Age group	Gender	Hamlet	Date	Age.gender	Age.hamlet	gender.hamlet	Age.gender.hamlet
		% (*p*)	% (*p*)	% (*p*)	% (*p*)	% (*p*)	% (*p*)	% (*p*)	% (*p*)
A. Clinical Phenotypes									
Number of visits Pf+	PFA	**2.3**	**0.6**	**1.9**	**6.3**	0.11 (0.009)	0.69 (0.001)	0.15 (0.003)	0
Proportion of visits Pf+	PPFA	**1.8**	**0.5**	**1.5**	**5.0**	0.13 (0.027)	**0.51**	**0.12**	0.26 (0.041)
Number of non-malaria fever visits	NMF	**0.8**	**0.2**	**0.8**	**2.6**	**0.03**	**0.35**	0.03 (0.025)	**0.03**
Number of visits Pv+	PVA	**4.4**	**0.3**	**0.4**	**4.2**	0.3 (0.003)	**1.4**	0.2 (0.027)	0
Proportion of visits Pv+	PPVA	**3.5**	**0.2**	**0.4**	**3.3**	0.2 (0.003)	**1.1**	0.14 (0.027)	0
B. Parasite Biological Phenotypes									
Pf max parasite density	mx-PFD	**17.7**	0.5 (0.022)	0.8 (0.014)	**10.1**	0	2.0 (0.004)	0	0
Pf parasite densities	PFD	**11.2**	0	0	**7.2**	0	0	0	0
Pv max parasite density	mx-PVD	**6.2**	0	0.6 (0.12^a^)	**8.8**	0	5.1 (0.003)	0	0
Pv parasite densities	PVD	**3.3**	0	3.0 (0.001)	5.9 (0.011)	0	0	0	0

In parentheses, *p* is the *p-value*; in bold, *p-value*<10^−3^.

a Retained because of significant interaction with Age.

The residual variance not explained by the “environmental” factors was generated. Because a non-normal error distribution was used, Pearson rather than standardized normal residuals were generated. For the phenotypes concerning number and proportion of episodes, the sum of the residuals per person were then calculated and used in the genetic analysis. For “all parasite densities”, all residual values for any individual (who had repeated parasite density measures) were then used in the genetic analysis. Only phenotypes from individuals for whom family structure was available were analysed for heritability.

### Genetic and house data analysis

To determine the contribution of genetic factors to clinical episodes and parasite density phenotypes, we evaluated the *h^2^* of “single value” phenotypes (number/proportion of cases and maximum density values) by using the SOLAR software package (version 2.1.4) [18]. SOLAR performs a variance components analysis of family data that decomposes the total variance of the phenotypes into components that are due to genetic (polygenic) (*h^2^*), individual or environmental (*e^2^*) and house (*c^2^*) effects. We tested for the heritability of the phenotype by comparing likelihood between the reduced model, where total variation is due to environmental variation only, and the full model where total variation is composed of environmental and genetic effects estimated from the genetic relationship coefficient of each pair of individuals. When the null hypothesis was rejected, heritability (*h^2^*) was then estimated as the percentage of genetic variance of the total. Although SOLAR can additionally incorporate measured covariates (e.g. explanatory variables), a normal distribution is assumed. For this reason we took into account the contribution of such variables in an initial statistical analysis (section above) and generated residual value for the phenotypes. The relative contribution of genetic factors to phenotype variation is then estimated by the heritability (*h^2^*), defined by the ratio of genetic variance component to the residual phenotypic variance [18]. As several phenotypes showed residual kurtosis of more than 0.8, *tdist* option, which creates an extra parameter in the model to describe the distribution of the phenotype, was applied in all analyses. An additive model, which is a general model, making no assumptions of the dominant or recessive nature of the gene, was used to avoid multiplying tests.

Household can confound the estimation of the genetic contribution to a trait, because related individuals often live in the same house. A household or shared environment effect can be added by an additional variance component with a coefficient matrix (H) whose elements (house) are 1 if the relative pair in shares the environmental exposure or 0 otherwise. Genetic effect (i.e. heritability) is estimated using matrix of correlation coefficients for identity by descent (IBD) allele sharing in various types of family relative pairs, whose elements (phi2) provide the predicted proportion of genes of the whole genome that a pair of individuals share at least 1 allele [18]. Table 4 shows the relationship of the house elements and phi2 elements. Even for the most closely related individual pairs (full sibs, parent-offspring), 38% live in different houses. In SOLAR we first included the house effect in the model. If the house effect was not significant (p value>0.05), we excluded it from the model for estimation of heritability.

**Table 4:** Number of pairs of individuals as defined by genetic relationship (phi2) and household relationship.

phi2	house				Total	Relationships
	0 (not shared)	%	1 (shared)	%		
0	448758	99.9	494	0.1	449252	Unrelated
0.625	0	0	2	100	2	Parent offspring from inbred family
0.5	2065	38.3	3331	61.7	5396	Full-sibs, parent offspring
0.375	3	50	3	50	6	Half-sib and first cousin, half-sib and half-avuncular
0.3125	2	100	0	0	2	Half-sib and half-first cousin
0.25	3974	92	344	8	4318	Half-sibs, avuncular, grandparent, first cousin
0.1875	14	100	0	0	14	First cousin, once removed from inbred family
0.125	3788	98.4	61	1.6	3849	First cousin, half-avuncular, grand-avuncular
0.09375	1	100	0	0	1	Second cousin from half-sib marriage
0.0625	1829	99.8	3	0.2	1832	Half-first cousin, half-grand-avuncular, great-grand-avuncular, great-great grandparent
0.03125	505	100	0	0	505	Second cousin
0.015625	8	100	0	0	8	Half-second cousin
Total	460947	99.1	4238	0.9	465185	

For the phenotypes for which there were multiple residual values (i.e. parasite densities for both *P. falciparum* and *P. vivax*), we evaluated heritability by using the classic repeated measures model (from the “animal model”) [19], where a permanent environmental effect is created for each individual. Thus the following model is fitted: y = [X*b*]+Z*a*+Z*pe*+Z*h*+*e* where y is the residual parasite density value, *b* is the fixed effects vector (here already taken into account in the first statistical analysis), *a* is the additive genetic effects vector, *pe* is the permanent environment effects vector (of each individual), *h* is the common house effect vector and *e* is the residual effects vector; × is the design matrix relating observations to fixed effects and Z are design matrices relating observations to random effects. The model was fitted using ASREML vers. 2 [19]. The total phenotypic variance is therefore V_P_, which is partitioned into V_A_, additive genetic variance, V_PE_, variance due to permanent environmental effects, V_H_, common-house variance and V_R_, residual variance. Heritability (*h^2^*) is again V_A_/V_P_.

### Genotyping

#### Microsatellite Genotyping

DNA was extracted from 10 mL venous blood or 0.5 mL capillary samples and randomly amplified using a primer extension pre-amplification method [20]. Genotyping of microsatellites (Linkage Mapping Set v2.5- MD10) was carried out using a ABI377 system (Applied Biosystems, Foster City, USA). The microsatellite genotyping results were here used for pedigree verification.

Genotyping results were checked for excess homozygotes or excess heterozygotes assuming Hardy-Weinberg equilibrium. Markers that showed excessive homo- or heterozygosity were re-examined. Mendelian inheritance within families was confirmed using the PedCheck program [21] and inconsistencies resolved by re-examination of the raw data and re-genotyping where necessary. After Mendelian inheritance was resolved, unlikely genotypes were detected by the program MERLIN [22]. Suggested microsatellite genotyping errors were corrected by re-examination of the raw data and re-genotyping where necessary.

#### Duffy blood group negative mutation (the DuffyT-46C)

We genotyped the Duffy T-46C SNP (rs2814778) [23] by TaqMan assay using ABI Prism 7000 Sequence Detection System with recommended protocol (TaqMan Universal Master Mix, 1 ng genomic DNA, 1× primer and probe mix). Primers and probes were Duffy F primer: CTGATGGCCCTCATTAGTCCTT, Duffy R primer: GCTGGGACGGCTGTCA, Duffy T allele: 5′VIC–CTTCCAAGATAAGAGCC, Duffy C allele: 5′FAM–CCAAGGTAAGAGCC.

## Results

From 1998 to 2005 (7 years) there were 19,162 independent clinical presentations by 2,545 individuals (Range 1–51 per person; mean 7.5 and median 5). Of these 2,430 were positive for *P. falciparum*, presented by 1,120 individuals (Minimum of 1 and a maximum of 13 positive presentations per person; mean 2.2 and median 1.5). There were 1,280 cases of *P. vivax*, presented by 636 individuals (Minimum of 1 and a maximum of 13 positive presentations per person; mean 2.0 and median 1), 30 cases of *P. malariae* and 11 cases of *P. ovale*. There were reliable *P. falciparum* trophozoite density data for 1,795 slides from 949 individuals and for 975 slides from 517 individuals for *P. vivax*; mixed infections were excluded. Only 38 *P. falciparum-P. vivax* and 3 *P. falciparum-P. malariae* mixed infections were observed. A total of 3,710 infections by any malaria parasite spp. were presented by 1,370 individuals presented with infection at any time over the period of study.

Of the 2,304 individuals for whom family structure was established, 2,018 came at least once to the clinic presenting a total of 16,926 visits. There were 2,179 cases of *P. falciparum* presented by 996 individuals and 1,118 cases of *P. vivax* by 555 individuals. There were 36 mixed *P. falciparum*-*P. vivax* infections and 3 mixed *P. falciparum-P. malariae* infections. Excluding mixed species infections, trophozoite density data was available for 857 individuals for *P. falciparum* and 470 for *P. vivax*. Table 2 records the samples used for the statistical analyses and the number for whom pedigree information was available and hence used for the heritability analyses. Table 3 shows the significant “environmental” factors in the statistical analyses from which residual values were generated for use in the heritability analyses and presents the percentage of variation explained for the given phenotype by each environmental variable. Date and age group have notably important influence on malaria parasite phenotypes.

### Correlation between phenotypes studied

Table 5 shows pair-wise correlation between the phenotypes studied. Of particular interest are (i) Number of non-malaria fever is strongly negatively correlated with the number of presentations with either *Plasmodium* spp. and has a higher negative correlation coefficient with the number of clinical *P. falciparum* attacks (R =  −0.78) than of *P. vivax* (R =  −0.43), (ii) Number of clinical *P. vivax* attacks shows a highly significant positive correlation with maximum or overall *P. falciparum* parasitemia (R  =  0.12 and 0.05, *p* =  0.0003 and 0.005 respectively) but negligible or no significance with *P. vivax* parasite density phenotypes (Maximum: R  =  0.1 *p* =  0.02, not significant after Bonferroni's correction and overall: R = 0.07 *p* =  0.25), (iii) Maximum *P. falciparum* density shows a positive correlation with maximum *P. vivax* density (R  =  0.22, *p* = 0.0002). Not surprisingly, the proportion of presentations with *P. falciparum* were very strongly correlated with the number of presentations with *P. falciparum* (R>0.90) and yielded very similar results in the statistical analyses. The same was true for *P. vivax*. These two proportional phenotypes yielded no additional information and were thus later removed from the final correlation analyses to reduce the overall number of pair-wise comparisons made.

**Table 5:** Pair-wise correlation between phenotypes studied.

Phenotype Acronym	PFA	NMF	PVA	mx-PFD	PFD	mx-PVD	
NMF	**−0.78**						corr coef.
	**<10^−4^**						*p-value*
	2544						n
							
PVA	0.003	**−0.43**					corr coef.
	0.88	**<10^−4^**					*p-value*
	2544	2544					n
mx-PFD	0.08	**−0.12**	**0.12**				corr coef.
	0.14	**0.0002**	**0.0003**				*p-value*
	949	949	949				n
PFD	**−**0.06	**−**0.002	*0.05*	**0.59**			corr coef.
	0.81	0.08	*0.005*	**<10^−4^**			*p-value*
	949	949	949	949			n
mx-PVD	0.02	**−** *0.12*	*0.10*	**0.22**	*0.11*		corr coef.
	0.71	*0.0049*	*0.021*	**0.0002**	*0.014*		*p-value*
	517	517	517	285	285		n
PVD	0.10	**−**0.15	0.07	**0.17**	*0.10*	**0.63**	corr coef.
	0.58	0.1	0.25	**0.002**	*0.01*	**<10^−4^**	*p-value*
	517	517	517	285	285	517	n

PFA, Number of visits Pf+; NMF, Number of non-malaria fever visits; PVA, Number of visits Pv+; mx-PFD, Pf max parasite density; PFD, Overall Pf parasite densities; mx-PVD, Pv max parasite density; PVD, Overall Pv parasite densities. In bold, highly significant p value (≤10^−4^); in italic, significant p value that becomes not significant after Bonferroni correction for multiple testing (21 hypotheses tested).

### Estimation of heritability and house effect (Table 6)

**Table 6:** Contribution (%) of genetic (heritability, h^2^) and house (c^2^) effects to variability in malaria and non-malaria clinical and biological phenotypes of *P. falciparum* (Pf) and *P. vivax* (Pv).

Phenotype	Acronym	N	Genetic effect	House effect
			h^2^ (S.E.), *p*-value	c^2^ (S.E.), *p*-value
A. Clinical phenotypes				
Number of visits Pf+	PFA	2018	12.4 (5.7), 0.009	4.6 (2.9), 0.05 a
Number of non-malaria fever visits	NMF	2018	NE, NS	8.7 (2.9), 7 × 10^−4^
Number of visits Pv+	PVA	2018	20.8 (3.6), 4.6 × 10^−4^	NE, NS
B. Parasite biological phenotypes				
Pf max parasite density	mx-PFD	857	23.7 (7.9), 1.5 × 10^−4^	NE, NS
Pf parasite densities	PFD	857	20.4 (4.3)	NE, NS
Pv max parasite density	mx-PVD	470	19.1 (10.5), 0.02	NE, NS
Pv parasite densities	PVD	470	14.9 (4.6)	NE, NS

h^2^, variance due to genetics, c^2^, variance due to house effect, S.E. the standard error; NS, not significant (*p-value*>0.05); NE, not estimated.

a Retained because of marginal significance.

#### Clinical phenotypes

Heritabilities (*h^2^*) of the number of clinical cases of *P. falciparum* and *P. vivax* were 12.4% (s.e. 5.7) and 20.8% (s.e. 3.6) respectively. There was a significant house effect for the number of clinical cases of *P. falciparum* (4.6% P = 0.05) but not for *P. vivax*. Similar values were obtained when considering the proportion rather than the number of all visits that were due to *P. falciparum* and *P. vivax*. The total number of non-malaria visits was greatly influenced by house (8.7%, P = 7×10^−4^) but not significantly so by genetics (6.5%, P = 0.09). Exclusion of house from the heritability analyses increased the genetic contribution to the number of *P. falciparum* cases to 19.7% and to the number of non-malaria cases to 18%.

#### Parasite biological phenotypes

There was moderate heritability of parasite trophozoite density phenotypes for both *P. falciparum* (Max. 23.7% and overall 20.4%) and *P. vivax* (19.1% and 14.9%). There were no effects of house on these phenotypes.

#### Genetic and environmental contributions to parasite-related phenotypes

**Figure 1:**
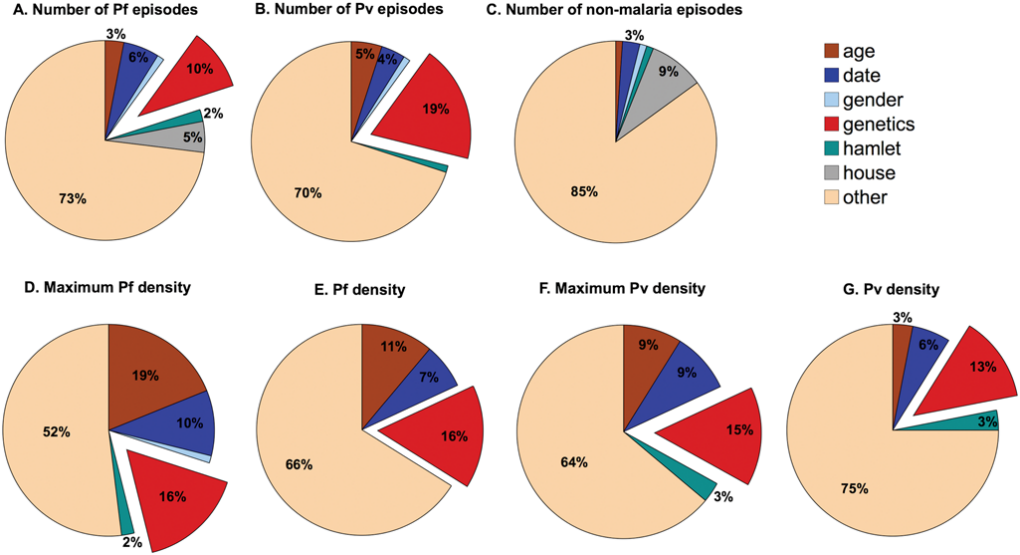
Proportions of variation explained by genetic heritability, house and environmental factors found to have a significant effect on the phenotype measured (Table 3&6). (A) Number of clinical episodes of *P. falciparum* (PFA); (B) Number of clinical episodes of *P. vivax* (PVA); (C) Number of non-malaria clinical episodes (NMF); (D) Maximum *P. falciparum* parasite density (mx-PFD); (E) Overall *P. falciparum* parasite density (PFD); (F) Maximum *P. vivax* parasite density (mx-PVD); (G) Overall *P. vivax* parasite density (PVD). Values of 1% or less not indicated numerically in the figure.

We have partitioned the total variation in the number of clinical malaria and parasite density phenotypes into its genetic, house and environmental components (Tables 3&6 and Figure 1). Of particular interest are (i) the significant effect of house on clinical *P. falciparum* phenotypes but the insignificant effect on clinical *P. vivax* phenotypes, (ii) the higher genetic effect for *P. vivax* clinical phenotypes than for *P. falciparum* and (iii) the very strong effect of age on *P. falciparum* parasite density.

#### Screening for Duffy negative blood group

We screened for Duffy negative blood group using polymorphism (DuffyT-46C) [23] in 377 unrelated individuals and did not find any allele associated with Duffy negative blood group phenotype in this population. Thus there is only a 2% probability of not finding the mutant allele if it is present at a frequency of only 1%.

## Discussion

Our clinical malaria longitudinal cohort study, conducted in the western province of Thailand, focused on analyses of the number of clinical attacks and parasite density of both *P. falciparum* and *P. vivax* after controlling for environmental effects. For all malaria phenotypes considered, with the exception of *P. falciparum* maximum parasite density, genetic effects explained more of the phenotypic variability than any environmental effect. The parasite species-specific genetic contributions varied according to the phenotype, but were similar for both species. Although direct comparison of genetic contributions between sites is confounded by the differing importance of environmental factors, it is notable that the total variation in number of malaria species episodes explained by host genetic factors was similar in both this study and the previous study in Sri Lanka [15]: 10% and 19% of the total variation in the number of clinical *P. falciparum* and *P. vivax* episodes respectively, compared to 15% for either species in Sri Lanka. Moreover, the host genetic contribution to *P. falciparum* parasite density was similar in both sites; only the genetic contribution to *P. vivax* parasite density was found to be different here (13%) as compared to 0% in Sri Lanka.

One pertinent difference between the two studies is the relative importance of house as a covariate. In the Sri Lanka study, house had a more significant influence on *P. vivax* than on *P. falciparum*. In our study, there was only a marginal effect of house on *P. falciparum* and no effect on *P. vivax*. Such clustering of *P. falciparum* cases has been previously observed in Thailand [24], Sri Lanka [15] and Africa [16], [25]. The generally weaker effect of house here compared with Africa in particular [16] most likely reflects the different mosquito biting behaviour of the major vector species. In Africa, *Anopheles gambiae* and *An. funestus* are predominantly endophilic, whereas the major vectors here, *An. maculatus* and *An. minimus*, are exophilic. House structure and proximity to mosquito breeding and resting sites would therefore be expected to be less important in Thailand than in Africa.

The absence of clustering of *P. vivax* cases suggests that some additional factor impacts upon *P. vivax* compared with *P. falciparum*. Differential *Plasmodium* species-specific vectorial competence and behaviour could result in differences in house clustering. An alternative explanation may reflect malaria species interactions. The capacity of one *Plasmodium* species to suppress another within the human is to some extent dependent on the infection status of each species, where both *P. falciparum* and *P. vivax* can dominate the infection. However, *P. falciparum* generally dominates when present [26], [27]. In this study site, a large number of *P. vivax* cases occurred following treatment of *P. falciparum* [17], as has been previously found in Thailand [28]. Thus *P. vivax* infections may be likewise clustered but do not become patent because of suppression by *P. falciparum*. The overlapping age-incidence curves of both parasite species observed here may generate more potential for species interactions than in Sri Lanka where peak incidence of disease does not overlap.

The strong negative correlation between the number of non-malaria fevers (NMF) and *P. falciparum* cases (PFA) suggests that illness due to non-malaria pathogens may protect from *P. falciparum* infection or disease and *vice versa*. The incidence of both diseases is concentrated annually in a very short time span, thus enabling such negative interactions. This is unlike the annual distribution of *P. vivax* cases (PVA) that is less concentrated into one peak, most probably because of its ability to relapse; this could then explain the lower negative correlation of PVA with NMF.

The positive correlation between the number of *P. vivax* episodes (PVA) and *P. falciparum* parasite density, especially maximum density (mx-PFD) (and to a lesser extent between PVA and mx-PVD) suggests that common mechanisms are involved in determining these phenotypes. To investigate this, we examined the effect of having a *P. vivax* episode during the first three years of study on the mx-PFD for each corresponding individual during the last 4 years of the study, taking into account environmental parameters. Those previously infected with *P. vivax* (1998–2000) had higher mx-PFD in 2001–2004 (P<0.001). They also had a greater number of *P. vivax* episodes (P<0.001), but did not have significantly different mx-PVD from those not previously infected with *P. vivax* (P = 0.16). This suggests that common mechanisms do govern susceptibility to infection by *P. vivax* and control of *P. falciparum* density but not control of *P. vivax* density. There was, however, a strong positive correlation between maximum *P. vivax* parasite density (mx-PVD) and mx-PFD. Given the differences in red blood cell (rbc) tropism (reticulocytes for *P. vivax* and all rbc types for *P. falciparum*), although blood disorders may be implicated, the involvement of shared immunological mechanisms is likely. It has been suggested that the two species may negatively impact upon one another through the development of cross-species immunity [29] and there is evidence for species-transcending parasite density-dependent immune responses [30], [31]. Parasite density phenotypes were not influenced by house in Thailand, consistent with the hypothesis that although house can contribute to the tendency to become infected, it does not impact upon the parasite once the infection has started. For both species environment plays a relatively larger role in determining the probability of infection, and genetics in determining the outcome of infection.

To our knowledge, the only known human genetic influence on resistance to *P. vivax* infection is the effect of Duffy blood group antigen on the inability of *P. vivax* merozoites to invade Duffy negative red blood cells [32], [33]. The Duffy negative blood group is mostly found in West Africa [34]. However, emergence of the same null allele mutation (T-46C) [23] but on a different haplotype was reported in Papua New Guinea and which affects expression of the Duffy blood group [35] and leads to reduced *P. vivax* erythrocyte infection [36]. We could not identify this mutation in 377 unrelated individuals in our population. This screening gave the probability of 0.0005 to have this mutation at a frequency of 0.02 in our population (frequency found in Papua New Guinea). It is thus unlikely that this mutation is responsible for the genetic contribution found for *P. vivax* in this study, although we cannot exclude other mutations in *DARC* gene coding for Duffy blood group. Other inherited blood disorders occurring regionally, such as the thalassemias, HbE and Glucose-6-Phosphate Dehydrogenase may be involved in determining susceptibility to *P. vivax* infection (and *P. falciparum* parasite density). Numerous population studies have confirmed the important protective role of such genetic disorders [5], [6], [37], but have concentrated on severe malaria and *P. falciparum* even when studied in South-East Asia [38].

A recent study on heritability of *P. falciparum* phenotypes in Senegal found that parasite density was under a similar genetic effect to PFA (*h^2^* 21% *vs*. 28%) [39]. Here we again find a similar genetic effect on density and number of attacks (*h^2^* 20% *vs*. 20% [excluding house effect]). The similarity of these values is all the more remarkable given the very differing transmission intensities observed in the two areas and differing human genetic background (i.e. ethnicities). The marginally lower heritability of number of episodes in this study site likely reflects lower transmission intensity that has lead to increased heterogeneity in exposure. The difference in the contribution of genetics is, however, small given the very large difference in transmission intensity. The statistical analyses that take into account environmental factors prior to genetic analyses seemingly effectively account for differences in transmission heterogeneity (risk of being infected). The high similarity of the genetic contributions to the parasite density is independent of acquired immunity, accounted for by use of age. It should be noted, however, that there may be a genetic contribution to acquired immunity, as previously shown [40], [41], that we have not considered here. Exclusion of acquired immunity therefore focuses on the genetic contribution exerted through innate immunity pathways and red blood cell mutations known to have an impact on the parasite. The latter have been the subject of considerable study and major mutations differ in the Thailand and Senegal [3], [42]. It is therefore striking that despite such known differences, the heritability of parasite density should be so similar.

Finally, it is important to note that we estimated only the additive genetic effect. The non-additive genetic effect may have an important contribution to overall estimates of heritability. However, the study population is inbred, which could lead to bias in estimation of non-additive genetic effect: the chance to be homozygous at particular regions of the genome is higher than under panmixia. In addition, positive selection could lead to an increase in frequency of some polymorphisms or mutations, which would contribute to higher heritability of the traits than here estimated.

In conclusion, our study clearly shows that there are major human genetic effects influencing both clinical response to infection and parasite parameters describing the infection status of both *P. falciparum* and *P. vivax*. Although environmental factors also play some role, their importance varies according to the infection phenotype considered. Environment does seemingly play an important role in acquiring an infection, but genetics plays a determinant role in the outcome of an infection with either malaria parasite species prior to the development of immunity.
